# Mental well-being at the workplace

**DOI:** 10.4103/0019-5278.75691

**Published:** 2010

**Authors:** T. Rajgopal

**Affiliations:** Vice President, Medical and Occupational Health, Hindustan Unilever Limited, 165/166, Backbay Reclammation, Hindustan Lever House, Mumbai, India. E-mail: thirumalai.rajgopal@unilever.com

It is increasingly being recognized that the mental health of employees is a crucial determinant in their overall health and that poor mental health and stressors at the workplace can be a contributory factor to a range of physical illnesses like hypertension, diabetes and cardiovascular conditions, amongst others. In addition, poor mental health can also lead to burn-out amongst employees, seriously affecting their ability to contribute meaningfully in both their personal and professional lives.[1]

Data from different countries around the world indicate that mental health problems are a cause of a number of employees dropping out of work. In the Netherlands, around 58% of the work-related disabilities are related to mental health.[2] In the UK, it is estimated that around 30–40% of the sickness absence is attributable to some form of mental illness.[3]

Mental health problems have an impact on employers and businesses directly through increased absenteeism, negative impact on productivity and profits, as well as an increase in costs to deal with the issue.[4] In addition, they impact employee morale adversely.

Work-related stress is a major cause of occupational ill health, poor productivity and human error. This means increased sickness absence, high staff turnover and poor performance in the organization and a possible increase in accidents due to human error. Work-related stress could also manifest as heart disease, back pain, headaches, gastrointestinal disturbances or various minor illnesses; as well as psychological effects such as anxiety and depression, loss of concentration and poor decision making.[5]

Stress is the adverse reaction people have to excessive pressures or other types of demands placed upon them. There is a clear distinction between pressure, which can be a motivating factor, and stress, which can occur when this pressure becomes excessive.

Some occupations are at more risk of mental health problems than others. A study in the Netherlands mapped skill levels against the pace of work to have an idea about the risk for stress levels and mental ill health for different occupations. Higher stress levels correlated with a higher risk for mental ill health.[6]

Figure 1 maps the risks for stress and mental ill health for a range of occupations, based on work pace and skills.

**Figure 1:**
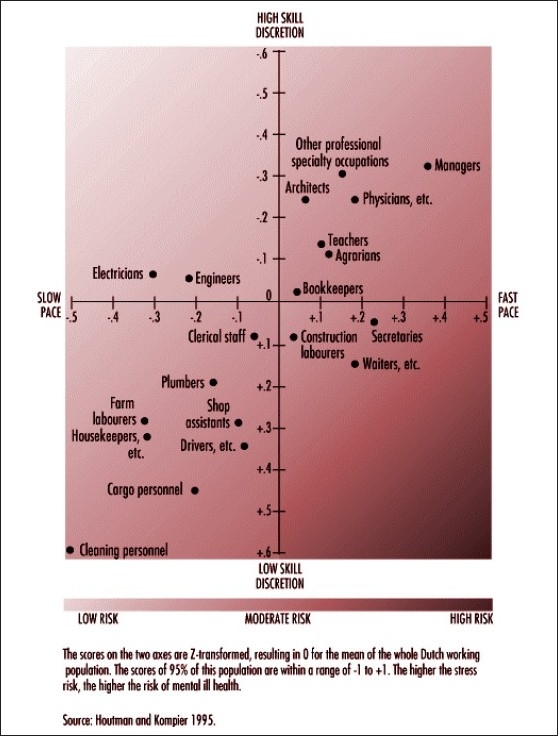
Risk for stress and mental ill health for different occupational groups, as determined by the combined effects of work pace and skill discretion (Source: ILO Encyclopaedia of Occupational Health and Safety 4^th^ Edition, Edited by Jeanne Mager Stellman, ILO Geneva, 1998)

## MEASURING AND CONTROLLING WORK - RELATED STRESS AND IMPROVING MENTAL WELL-BEING AT THE WORKPLACE

A tool to evaluate the level of work-related stress and the measures to be taken thereof to control the same have been extensively used.[7–10] This tool which is known as the Work Stress Scale (WSS) allows individuals to assess for themselves the degree of stress faced in the following broad domains:

- relationship problems with superiors;
- bureaucratic constraints;
- work family conflict;
- relationship problems with colleagues;
- performance pressure and
- poor job prospects.

### Relationship problems with superiors

The most common reason for office stress is dealing with difficult boss. But this may be far easier to solve by improving communication skills. Having a sincere conversation may make a difference. Sometimes, the boss may set unreal targets, where an honest discussion can bring out what deadlines can be met.

Tasks that are not part of an employee role or skill set can also cause stress. Companies often make employees multitask but this could potentially affect their ability to deliver. Communicating with superiors about this matter at the earliest is the best way to resolve this. One area that presents an opportunity for conflict for the personality-disordered individual concerns the hierarchical nature of organizations.[11]

### Relationship problems with colleagues

Another reason could be difficult colleagues or co-workers. Dealing with a difficult co-worker can be a bit more difficult as their performance is often pitted against oneself. This again has to be resolved by an amicable discussion, concluded by a mutual agreement. One can explain to the colleague as how a team can have far more benefits than indulging in rivalry. But if things are getting out of hand, it should be brought to the notice of the superior concerned.

### Work family conflict

Families are struggling to cope with an increasingly complex world. Individuals are struggling to find the right balance between work and family responsibility.[12] Domestic issues can affect work where balancing work and home by allotting adequate time for both can help reduce stress.

### High demand for performance

Unrealistic expectations, especially in the time of corporate reorganizations, which, sometimes, puts unhealthy and unreasonable pressures on the employee, can be a tremendous source of stress and suffering. Increased workload, extremely long work hours and intense pressure to perform at peak levels all the time for the same pay, can actually leave an employee physically and emotionally drained. Excessive travel and too much time away from family also contribute to an employee’s stressors.

### Job insecurity

Organized workplaces are going through metamorphic changes under intense economic transformations and consequent pressures. Reorganizations, takeovers, mergers, rightsizing and other changes have become major stressors for employees, as companies try to live up to the competition to survive. These reformations have put demand on everyone, from a CEO to a line manager.

### Bureaucratic constraints

Organizational size and bureaucratic systems have certain rules and regulations, which are inherent parts of the system to serve as checks and balancing forces.

However, they are likely to serve as constraints and stress for managers. Other job stressors include uncomfortable working conditions, job overload, lack of control over the work process and sheer monotony.[13] Decreasing work role ambiguity would reduce job strain and work-related psychological disorders including anxiety disorders.[14]

Companies would do well to address mental wellness at the workplace through a clearly articulated workplace policy on mental health. A prototype of such a policy encompassing the vision, the values and principles and the objectives is appended [Table 1].

**Table 1:** Sample workplace mental well-being policy

Vision
__________________________(*Company’s name*) is committed to ensure the health and well-being of employees at the workplace. The company recognizes that the mental health of employees is a crucial determinant in their overall health and that poor mental health and stressors at the workplace can be a contributory factor to a range of physical illnesses like hypertension, diabetes and cardiovascular conditions, amongst others. In addition, poor mental health can also lead to burn-out amongst employees seriously affecting their ability to contribute meaningfully in both their personal and professional lives. We will assess and respond quickly to the needs of employees with mental health problems.
Values and principles
Employees are our most valuable asset. We will provide adequate support services to employees to benefit from counseling and other interventions to ensure positive mental health. We will ensure that employees have access to treatment for mental health problems where needed. We will periodically assess workplace stress through appropriate surveys conducted ethically. Based on our internal workplace stress surveys, we will initiate programs to cover the following: ♦ information and awareness campaigns; ♦ employee and manager training; ♦ promoting mental health through our “agile working” initiative; ♦ addressing harassment at the workplace; ♦ specific interventions for identified stressors at the workplace; ♦ training in resilience techniques and coping strategies; ♦ specifically address the prevention, identification and management of depression and anxiety at the workplace; ♦ supporting individuals with mental health problems; and ♦ recruitment and retention practices which do not discriminate against people with mental health problems.
Objectives of the policy
We will evaluate and control organizational factors that could potentially contribute to work-related stress. We will implement stress and burn-out prevention and management programs for employees at risk as well as early detection and support programs to deal with alcohol or mental health problems once they have occurred to ensure that an employee can contribute meaningfully to himself, his family and at the workplace. We seek to enhance the productivity of the business by providing adequate support to employees following any mental health deviations. We will minimize disability amongst employees by providing specific interventions against conditions like depression and anxiety.

The development and implementation of a workplace mental health policy and program will benefit the health of employees, increase the productivity of the company and will contribute to the well-being of the community at large. It has been found that psychosocial intervention courses along with stress management training and health promotion interventions have a positive impact on mental well-being.[15]

A healthy population is an economically productive population and it is in the benefit of companies to safeguard public health. Given the heavy contributions of the private sector to the economy, employee wellness programs are not only a strategic priority for India but also an economic imperative for corporations.[16]
